# CRISPR/Cas9-mediated *VDR* knockout plays an essential role in the growth of dermal papilla cells through enhanced relative genes

**DOI:** 10.7717/peerj.7230

**Published:** 2019-07-03

**Authors:** Ye Gao, Miaohan Jin, Yiyuan Niu, Hailong Yan, Guangxian Zhou, Yulin Chen

**Affiliations:** 1 Department of Neurology, Institute of Brain Science, Medical School, Shanxi Datong University, Datong, China; 2 Shanxi key Laboratory of Inflammatory Neurodegenerative Disease, Institute of Brain Science, Shanxi Datong University, Datong, China; 3 College of Animal Science and Technology, Northwest A&F University, Yangling, China

**Keywords:** Cashmere goat, CRISPR/Cas9, *VDR*, DP cells

## Abstract

**Background:**

Hair follicles in cashmere goats are divided into primary and secondary hair follicles (HFs). HF development, which determines the morphological structure, is regulated by a large number of vital genes; however, the key functional genes and their interaction networks are still unclear. Although the vitamin D receptor (*VDR*) is related to cashmere goat HF formation, its precise effects are largely unknown. In the present study, we verified the functions of key genes identified in previous studies using hair dermal papilla (DP) cells as an experimental model. Furthermore, we used CRISPR/Cas9 technology to modify the *VDR* in DP cells to dissect the molecular mechanism underlying HF formation in cashmere goats.

**Results:**

The *VDR* expression levels in nine tissues of Shaanbei white cashmere goats differed significantly between embryonic day 60 (E60) and embryonic day 120 (E120). At E120, *VDR* expression was highest in the skin. At the newborn and E120 stages, the VDR protein was highly expressed in the root sheath and hair ball region of Shaanbei cashmere goats. We cloned the complete CDS of *VDR* in the Shaanbei white cashmere goat and constructed a *VDR*-deficient DP cell model by CRISPR/Cas9. Heterozygous and homozygous mutant DP cells were produced. The growth rate of mutant DP cells was significantly lower than that of wild-type DP cells (*P* < 0.05) and *VDR* mRNA levels in DP cells decreased significantly after *VDR* knockdown (*P* < 0.05). Further, the expression levels of *VGF, Noggin, Lef1,* and *β-catenin* were significantly downregulated (*P* < 0.05).

**Conclusions:**

Our results indicated that *VDR* has a vital role in DP cells, and that its effects are mediated by Wnt and BMP4 signaling.

## Introduction

The Vitamin D receptor (*VDR*) plays a key role in hair growth, and human *VDR* mutations can lead to alopecia. *VDR* knockout mice exhibit hair loss and poor hair growth (Rosen et al., 1979; Sakai & Demay, 2000). *VDR* interacts with the WNT, HH, and TGF signaling pathways to promote hair growth (Demay et al., 2007; Pálmer et al., 2008a; Palmer et al., 2008b; Teichert, Elalieh & Bikle, 2010; Demay, 2012; Lisse et al., 2014). Although the effects of *VDR* on hair growth have been analyzed extensively in humans and mice, its mechanism of action in cashmere goats remains unclear. Cashmere has great economic value, and the formation and development of hair follicles (HF) contributes to high cashmere production in Shaanbei white cashmere goats.

Using an RNA-seq approach, we previously found that VDR expression is significantly higher in HF at embryonic day 120 (E120) and in newborn (NB) individuals than at E60 in Shaanbei white cashmere goats. (Gao et al., 2016). Thus, *VDR* might play a key role during the embryonic period in cashmere goats. However, the regulatory mechanism underlying its effects is still unclear. Functional studies of *VDR* could lay a solid foundation for molecular breeding of *VDR*-modified cashmere goats with improved cashmere production.

Compared to zinc-finger nuclease and ES cell targeting and transcription activator-like effector nucleases (TALENs), the clustered regularly interspaced short palindromic repeats CRISPR-associated 9 (Cas9) system is a more precise gene editing technique and has been widely used in cashmere goats (Wang et al., 2015; Wang et al., 2016b). In this study, the CRISPR/Cas9 system was used to generate a modified cell line to investigate the functions of *VDR*. In particular, dermal papilla (DP) cells play a central regulatory role in the development of HFs (Jahoda, Reynolds & Oliver, 1993; Driskell et al., 2011; Aoi et al., 2012a; Aoi et al., 2012b). We created a *VDR*-knockout DP cell line using CRISPR/Cas9 and compared its characteristics with those of negative control DP cells (wild type). Using this system, we proposed several molecular mechanisms that govern the effects of *VDR* on HF growth in Shaanbei white cashmere goats.

## Materials & Methods

### Animals

All studies and methods were conducted in accordance with approved guidelines of the Experimental Animal Committee of the Northwest A&F University (Approval ID: 2014ZX08008-002).

### Experimental materials

PGL3-U6-sgRNA-PGK-puromycin and pST1374-NLS-flag-linker-Cas9 were donated by Prof. Huang Xingxu from Shanghai Tech University. Experimental animals were provided by the Shaanbei White Cashmere Goat Original Seed Farm, Shaanxi Province.

### *VDR* expression in Shaanbei white cashmere goats during the fetal period

Skin, heart, liver, spleen, kidney, stomach, brain, muscle, and small intestine tissue samples were collected from Shaanbei cashmere goats at E60 and E120. Tissues were cryopreserved in liquid nitrogen and stored prior to use. Total RNA was extracted and *VDR* expression was detected in each tissue by quantitative real-time PCR (qRT-PCR).

### Immunohistochemical *VDR* staining in Shaanbei White Cashmere Goat Skin Tissue at different stages of Gestation

Shaanbei white cashmere goats at E60, E120, and NB were handled as described previously (Gao et al., 2016). Lateral skin tissues were paraffin-embedded and used for VDR immunohistochemical staining. Images of sections were obtained under a light microscope after drying.

### Construction of the *VDR*-deficient DP Cell Model

#### CDS region amplification of *VDR*

Vitamin D (1,25-dihydroxy vitamin D3) receptor (*VDR*) primers were designed according to predicted mRNA sequences available in NCBI for *Capra hircus VDR* (reference sequence XM_013963843.1) and *Pantholops hodgsonii VDR* (reference sequence XM_005981021.1). NHeI and BamHI sites were engineered on either side of the open reading frame. Primer sequences were as follows:

VDR-F: 5′-CTAGCTAGCATGGAGGCGACTGCGGCCAGCAGC-3′(NHeI);

VDR-R: 5′-CGCGGATCCTCAGGAGATCTCGTTGCCAAACAC-3′(BamHI).

Total RNA from skin tissues was extracted using RNAiso Plus (Takara, Japan) and a reverse transcription kit (Thermo Fisher Scientific, Waltham, MA, USA) was used to synthesize cDNA. Using this template, the CDS region of *VDR* was amplified using PrimeSTAR GXL DNA Polymerase (Takara, Kusatsu, Japan). The total volume of PCRs was 50 μL. The reaction conditions were as follows: 2 min at 98 °C, 34 cycles of 30 s of denaturation at 98 °C, 15 s of annealing at 60 °C, and 2.5 min of elongation at 68 °C, followed by a 10 min hold at 68 °C. PCR products were purified from 1% agarose gels. Adenine was added to 1 μg of the purified product and *VDR* segments were ligated into the pMD19-T (Simple) vector (Takara). Products were identified by PCR and commercial sequencing.

### Determination of sgRNA target sites

The CDS sequence of *VDR* was amplified and an sgRNA site was designed for insertion into the selected exon. Oligonucleotides for sgRNA plasmid construction (pGL3-U6-sgRNA-PGK-puromycin) are listed in Table 1.

**Table 1:** Target sequence for sgRNA and primers for plasmid construction.

sgRNA	Targeted sequence	Primers sequence
	CGGATCTGCGGGGTGTGCGG	F:ACCGCCGCACACCCCGCAGATCCG
		R:AAACCGGATCTGCGGGGTGTGCGG

### Construction of gene-modified DP cells

DP cells were separated as previously described. This method was established by He and Zhou in our laboratory (He et al., 2016; Zhou et al., 2017).

Gene editing experiments were performed by co-transfecting sgRNA expression vectors and pST1374-NLS-flag-linker-Cas9 into third-generation DP cells from cashmere goats using Lipofectamine 3000 (Thermo Fisher Scientific) for 48 h. Then, the cells were supplemented with optimal concentrations of blasticidin and puromycin (Thermo Fisher Scientific; 9 µg/mL and 0.8 µg/mL) for 48 h. Cells were seeded in 90-mm dishes at a density of 800 cells per well. After 7 d, cell colonies were removed with filter paper and seeded into 24-well plates for large-scale cultivation.

### Genotype identification of positive monoclonal cells

Genome extraction kits (CWBIO, Shenzen, China) were used to isolate the edited genome in DP cells. The region around the sgRNA target site was amplified using primer-F (5′-TGGCACCCGGAACCACAAA-3′) and primer-R (5′-CAGAGGAGGAAGAAACAGACC-3′) and products were purified from 1% agarose gels. As in our previous studies, genotype identification was performed by T7E1 (TOYOBO, Osaka, Japan) digestion and T-A monoclonal sequencing.

### Effects of *VDR* knockout in DP cells

Homozygous monoclonal cells with frameshift mutations were selected for further assessment. RT-qPCR primers for *VDR* are shown in Table 2. Homozygous mutant and negative control DP cells (wild type) were seeded into 6-well plates at a density of 8 ×10^4^ cells per well. Cells were collected for RNA extraction when they reached 90% confluency.

Reverse transcription and RT-qPCR were performed according to previously described methods (Gao et al., 2016). Cells in a single well were lysed to assess VDR** expression by western blotting following previously described methods (Wang et al., 2015). The intensity of VDR protein bands was analyzed using ImageJ.

**Table 2:** List of primers for qPCR.

Gene	Upstream primer	Downstream primer
*VDR*	CGACACCTACTCCGACTTCA	GGACGAGTTTCCAGAGAAGC

### Studying *VDR* function in DP cells

#### Effects of *VDR* knockout on DP cell proliferation

Negative control DP cells (wild type) and *VDR*-knockout DP cells were seeded into 24-well plates for 7 d at a density of 10^4^ cells per well. Each day, cells were collected and counted in a cell counting chamber. DP growth curves were created by plotting the number of cells over time from the initiation of the culture period. All experiments were performed in triplicate.

### *VDR* knockout and hair follicle development

Negative control DP cells (wild type) and *VDR* knockout DP cells were plated at a density of 8 × 10^4^ cells into 6-well plates. Total RNA was extracted when cells reached 80% confluency and the mRNA levels of genes regulating hair follicle growth (*Gli1*, *IGF-1*, β-catenin, *Lef1,* Noggin,** and *BMP4*) were assessed by RT-qPCR. Primer sequences are listed in Table 2. All experiments were performed in triplicate. Values were normalized against those for negative control DP cells (wild type). All data were analyzed using SPSS 17.0.

## Results

### VDR tissue expression profiling in shaanbei white cashmere goat during the fetal stage

**Figure 1:**
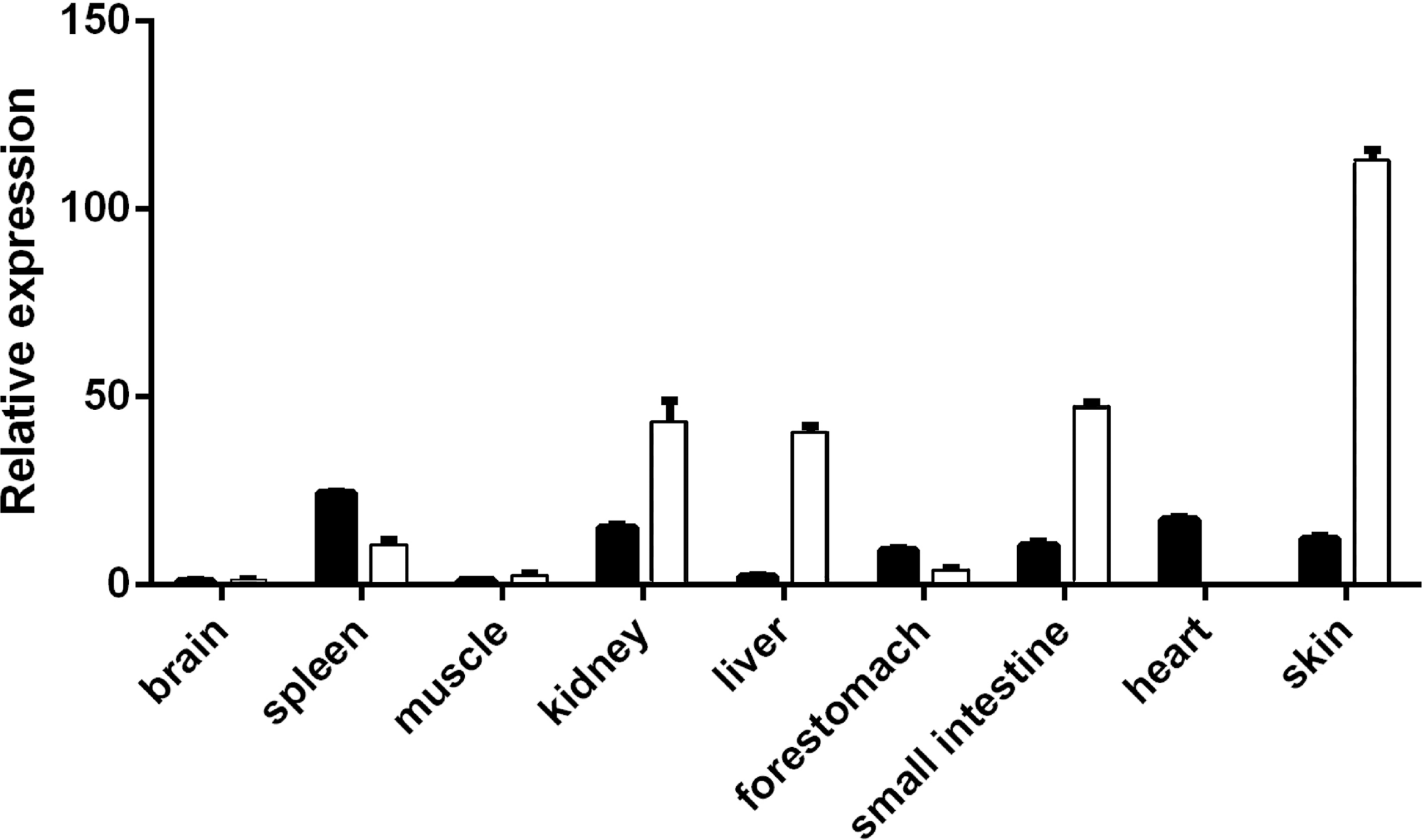
Expression spectrum of *VDR* in different tissues at E60 and E120. The black bar represents embryonic day 60 (E60). The white bar represents embryonic day 120 (E120).

**Figure 2:**
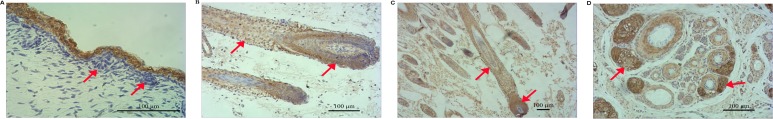
Distribution of *VDR* immunoreactivity in fetal goat skin. (A) E60: *VDR* shows intense immunostaining in the epidermis. Very little *VDR* immunoreactivity was detected in the placode (arrow) (200×). (B) E120: *VDR* immunoreactivity is predominantly detected in the ORS keratinocytes and the bulb of HF (arrow) (100×). (C) NB: In fully formed HF, *VDR* staining was present in the root sheaths and bulb cells, where immunoreactivity was particularly enhanced in the ORS keratinocyte and matrix zone (arrow) (40×). (D) NB: In the PHF and SHF, *VDR* staining was present in the sebaceous gland zone (arrow) (100×).

*VDR* expression levels in the skin, heart, spleen, kidney, brain, muscle, fore-stomach, and small intestine differed between the E60 and E120 stages. *VDR* levels were highest in the spleen at E60 and were higher in the kidney, liver, small intestine, and skin at E120 than at E60. However, at the E120 stage, *VDR* levels in the spleen, fore-stomach, and heart were lower than those at E60. The expression of *VDR* was the highest in the skin at E120 (Fig. 1), indicating an important role of *VDR* in skin tissues. The VDR distribution in the skin of goats at E60, E120, and NB was assessed by immunohistochemical analysis. VDR was periodically expressed in the skin at each stage and was most highly expressed in the epithelium. VDR expression was not evident in HFs at E60 but was expressed in the inner sheath, outer root sheath (ORS), and hair bulb of HFs at the E120 and NB stages, with the highest levels of expression in the follicle ORS, matrix (Mx), and sebaceous gland (Fig. 2).

### Construction of *VDR*-deficient DP cell models

**Figure 3:**
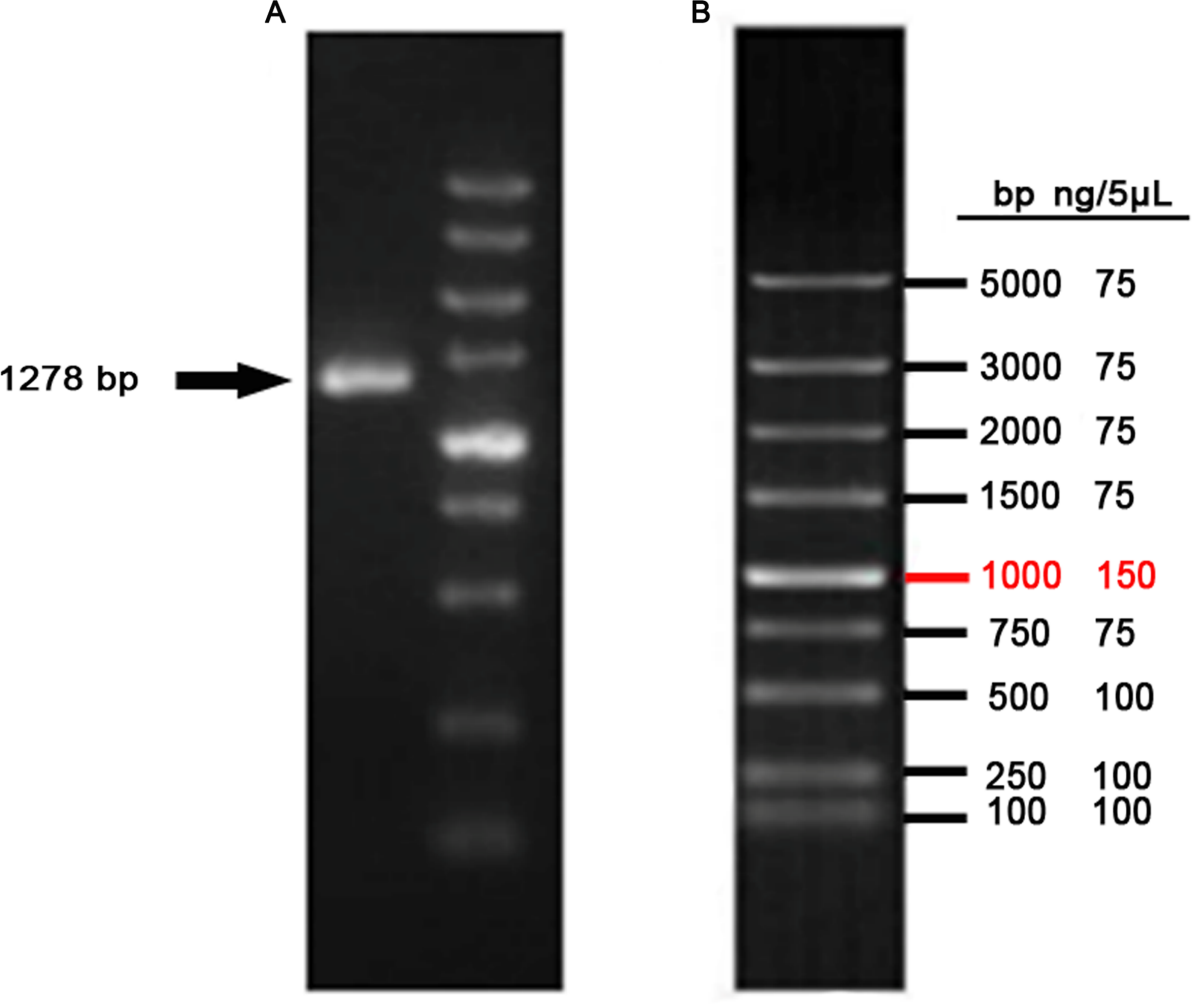
Amplification of *VDR*. (A) PCR product of *VDR.* (B) DL5000 DNA marker.

The CDS of *VDR* was PCR-amplified from a cDNA template, producing a 1278 bp product (Fig. 3). Products were sequenced and compared to publicly available goat *VDR* sequences in GenBank by Sanger sequencing.

**Figure 4:**
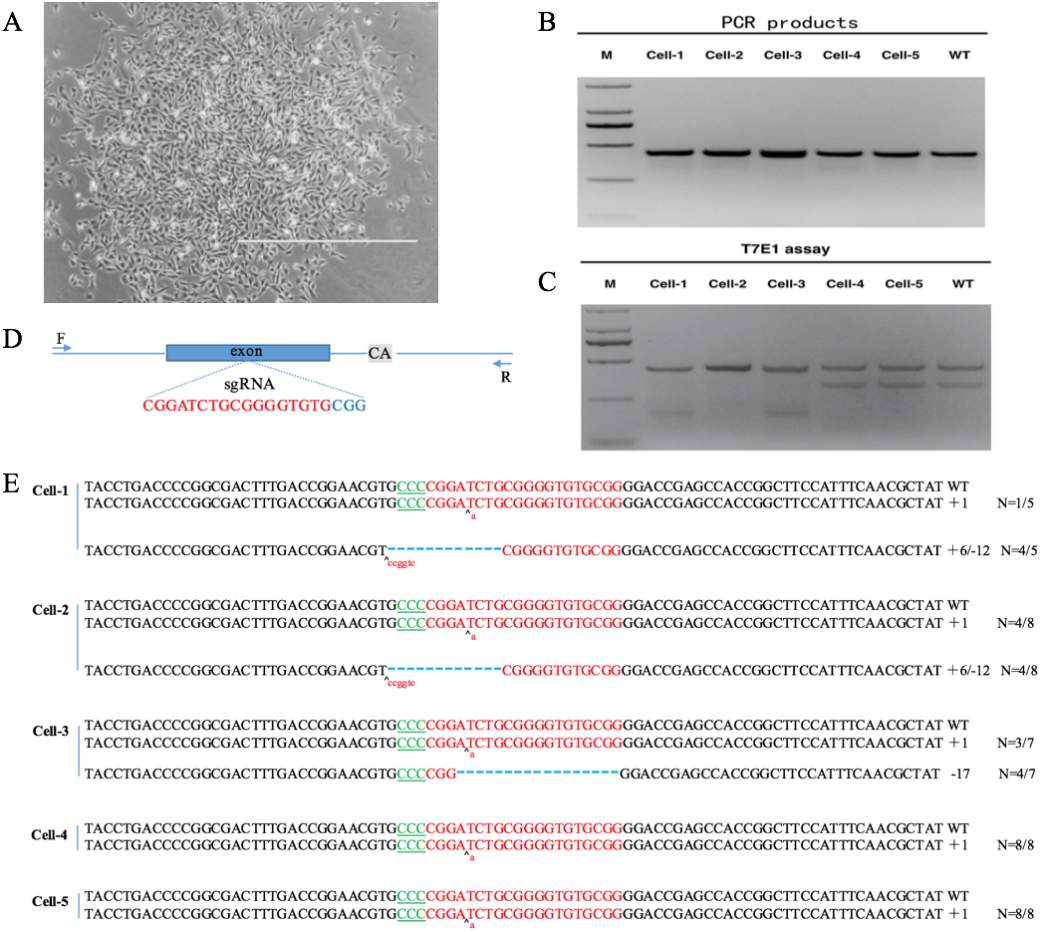
*VDR*-deficient DP cell obtain and identification. (A) Single cell clone (contrast microscope; Scale bar = 1,000 µm). (B) Detection of sgRNA: Cas9-mediated cell DNA PCR products (2000 DNA marker). (C) Detection of sgRNA: Cas9-mediated cleavage by T7E1 cleavage assay (2000 DNA marker). (D) Schematic diagram of genetic structures and targeting loci of sgRNA:Cas9; sgRNA targeting loci are highlighted in red; PAM sequences are highlighted in blue; gray background bases are original deletion bases in negative control cell(wild type). (E) Sequences of modified *VDR* by sgRNA: Cas9. Target sequences complementary to sgRNA are in green text; the mutations are red; insertions (+) and deletions (−) are shown.

After co-transfecting sgRNA expression vectors and pST1374-NLS-flag-linker-Cas9 into DP cells and antibiotics screening, cells were detached with 0.25% trypsin and 800 cells were seeded into 100-mm plates. Single cell colonies were removed with filter paper (Fig. 4A) and grown in 24-well plates. Six single cell clones were obtained.

DNA was extracted from positive clones. PCR and T7E1 cleavage revealed that the clones were positive (Fig. 4B). PCR products were cloned into the TA vector by Sanger sequencing. Because the negative control DP cells (wild type) also had a 2-bp deletion downstream of the sgRNA targeting site (Fig. 4C, Data S1, S2), every sample was cut by T7E1 (Fig. 4B). By Sanger sequencing, five positive single cell clones, named cell-1, cell-2, cell-3, cell-4, and cell-5, were identified. Cell-1, cell-2, and cell-3 were heterozygous and cell-4 and cell-5 were homozygous. Sanger sequencing showed that cell-4 and cell-5 had a single-base insertion in the sgRNA target site, leading to a frameshift mutation (Fig. 4D).

### Verification of *VDR* function in DP cells

**Figure 5:**
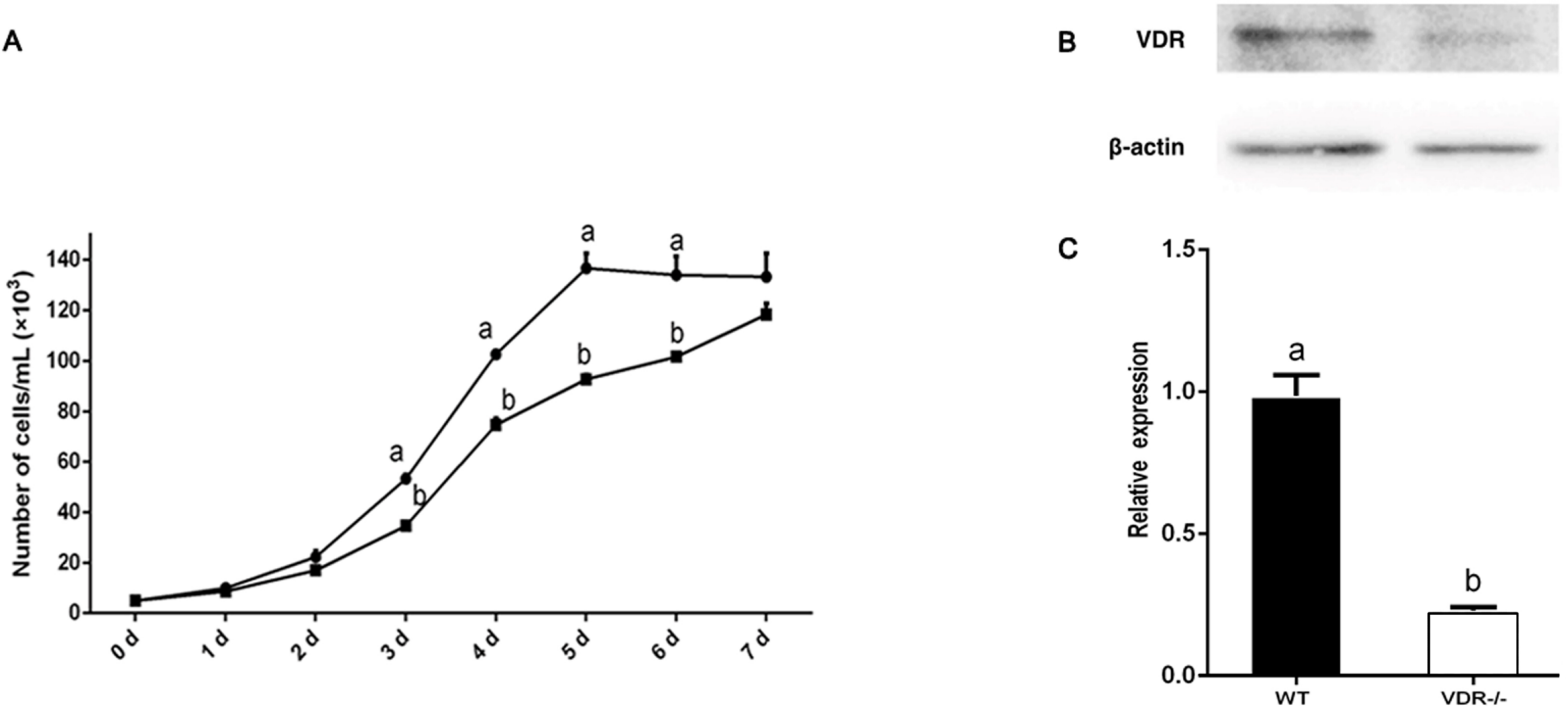
Verification of *VDR* function in DP cells. (A) Effects of *VDR* on the proliferation of DP cells. The black square represents *VDR*-/-cells and the black dot represents negative control (Wild type) (B) Western blot results showed the VDR protein level in single DP cell clone. (C) Relative expression of *VDR*-/-cells and negative control (Wild type).

Growth curves of cell-4 and negative control DP cells (wild type) were constructed to assess the effects of *VDR* knockout on DP proliferation. The growth of *VDR* knockout cells was significantly lower than that of the negative control DP cells (wild type) (*P* < 0.05) (Fig. 5A). Western blot analysis showed that the expression of VDR in DP cells decreased significantly after *VDR* knockdown (*P* < 0.05, Fig. 5B).

**Figure 6:**
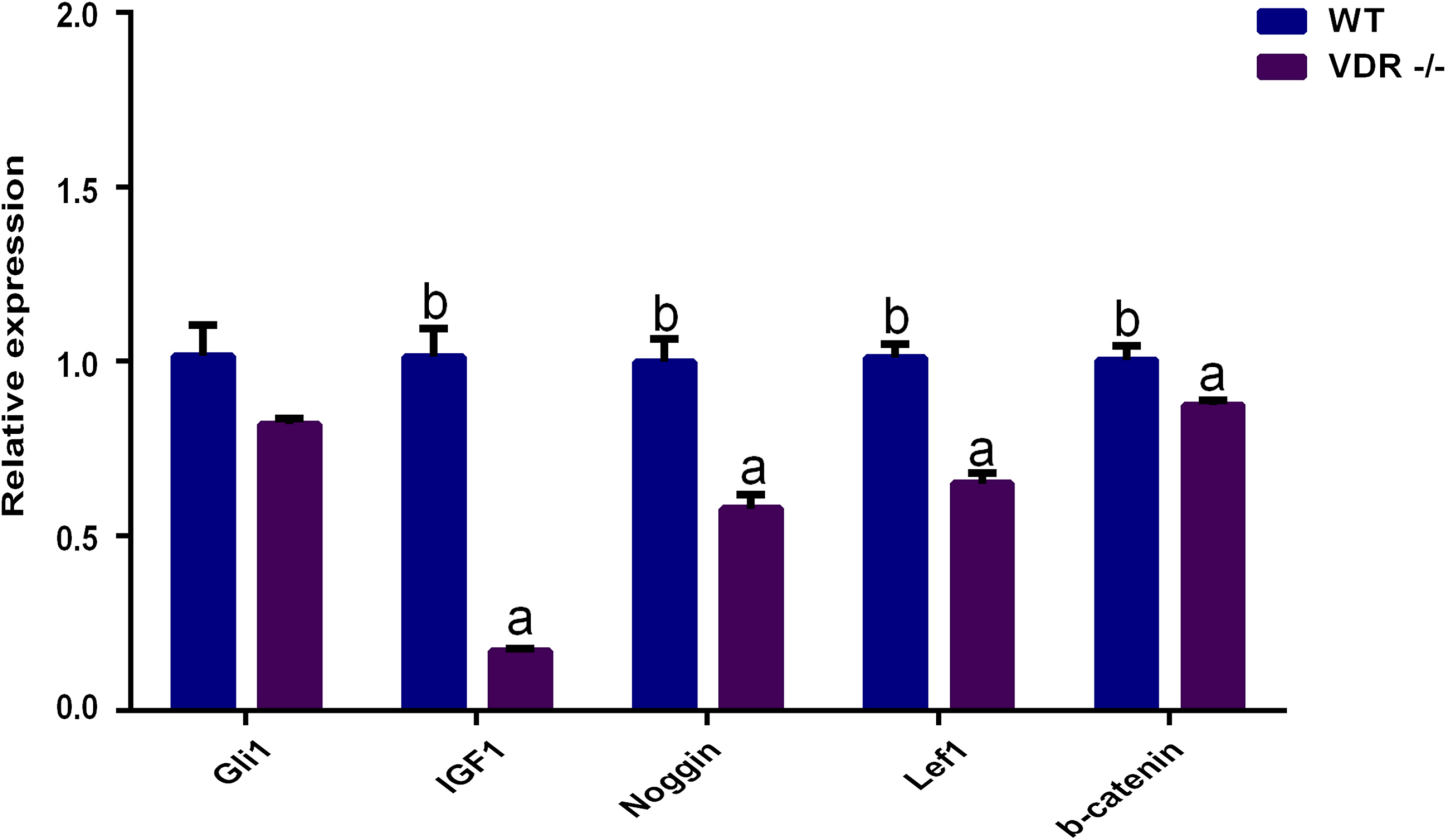
The relative genes expression at HF development in DP cells. The purple bar presents *VDR*-/-cells. The blue bar presents negative control (wild type).

RT-qPCR showed that the expression levels of *Gli1* decreased slightly in *VDR*-/- cells, but the difference compared to control DP cells was not significant (*P* > 0.05). The expression levels of *IGF-1,* Noggin*, Lef1,* and β-catenin in *VDR*-/-cells were significantly lower than those in the control group (*P* < 0.05, Fig. 6).

## Discussion

In this study, we silenced *VDR* expression using the CRISPR/Cas9 system in Shaanbei white cashmere goats to assess its effects on hair growth. We identified single cell clones harboring mutations with significantly lower growth rates compared to those in control groups. These results indicated that *VDR* knockout significantly inhibits DP growth. As DP cells play a central role in HF development and periodic growth (Jahoda, Reynolds & Oliver, 1993; Aoi et al., 2012a; Aoi et al., 2012b), we speculate that *VDR* is critical for these processes.

Additionally, the relative expression levels of related genes during HF development in DP cells were evaluated. *Gli1*, a target of HH, is a marker of quiescent HF stem cells (Brownell et al., 2011). Its expression was reduced in *VDR*-knockout mice, but HF growth could be recovered via Hedgehog signaling (Wang et al., 2000; Teichert, Elalieh & Bikle, 2010; Lisse et al., 2014). In contrast to these studies, we found that *VDR* knockout did not affect *Gli1* expression. This difference among studies may be attributed to the different cell lines assessed, as *VDR* may not regulate HH signaling in DP cells. These discrepancies require further analysis.

WNT, HH, and TGF/BMP are involved in the relationship between the epidermal and dermal cells of HFs and play an important role in HF development (Hardy, 1992; Botchkarev & Kishimoto, 2003; Sennett & Rendl, 2012). *IGF-1* links epidermal and dermal cells and promotes HF development and the maintenance of hair shaft growth (Su et al., 1999; Li et al., 2014). To assess the molecular mechanisms governing the ability of *VDR* to regulate DP growth, the expression of WNT, HH, and TGF/BMP were assessed by RT-qPCR in *VDR* knockout cells. We found that *Lef1* and β-catenin were significantly down-regulated following *VDR* knockout, suggesting that *VDR* is required for Wnt signaling in DP cells. In human keratinocytes, *VDR* knockout similarly inhibits the co-transcription of *Lef1* and β-catenin (Pálmer et al., 2008a; Palmer et al., 2008b). Wnt signaling is essential for the periodic growth of HFs as *Lef1* and β-catenin are critical to the differentiation of HF stem cells (DasGupta & Fuchs, 1999; Millar et al., 1999; Huelsken et al., 2001; Andl et al., 2002). In addition, Noggin has been shown to induce HF production by negatively regulating BMP4. Noggin is down-regulated in *VDR*-knockout mice (Botchkarev et al., 2002; Luderer, Gori & Demay, 2011) and we observed significantly reduced levels of *Noggin* expression in *VDR*-/- DP cells, indicating that *VDR* may similarly inhibit BMP4 signaling.

In summary, heterozygous and homozygous mutant DP cells were obtained using the CRISPR/Cas9 system. Future studies should evaluate whether VDR regulates HH signaling in DP cells or regulates HH signaling in other HFs as well as the precise regulatory mechanism by which *VDR* influences HF development and periodic growth. The *VDR*-modified cells developed in this study can be used to resolve these issues.

## Conclusions

In summary, studies of humans and mice have shown that *VDR* is essential for the initiation of the HF growth phase (Demay et al., 2007; Pálmer et al., 2008a; Palmer et al., 2008b; Demay, 2012). *VDR* knockdown using CRISPR/Cas9 in mouse myoblasts leads to hair loss (Wang et al., 2016a). We demonstrated that *VDR* plays an essential role in the growth of DP cells. *VDR* enhances Wnt signaling and promotes IGF-1 and Noggin expression. We speculate that *VDR* influences the development and periodic growth of HFs by influencing the mutual regulation of these pathways. Our newly generated cell model provides a basis for further studies of the development of HFs and breeding strategies aimed at increased cashmere production.

##  Supplemental Information

10.7717/peerj.7230/supp-1 Supplemental Information 1 Sequence of *VDR* CDS and negative cell control(wild type) Supplementary 1. Sequence of *VDR* CDS and the sgRNA position. Supplementary 2. Sequence of negative cell control (wild type).

10.7717/peerj.7230/supp-2 Supplemental Information 2 Effects of VDR on the proliferation of DP cells raw data Each data indicates the cell numbers of VDR ^−∕−^ cells and negative control (wild type) from day-1 to day-8 to show the cell growth curve. All the experiments were carried out in triplicate.

10.7717/peerj.7230/supp-3 Supplemental Information 3 Figure raw data (A) Raw figure of VDR PCR product (5,000-bp DNA marker). (B) Raw figure of detection of sgRNA: Cas9-mediated cell DNA PCR products (2,000-bp DNA marker). (C) Raw figure of detection of sgRNA: Cas9-mediated cleavage by the T7E1 cleavage assay (2,000-bp DNA marker). (D) VDR western blot raw figure. (E) *β*-actin western blot raw figure.

10.7717/peerj.7230/supp-4 Data S1 Cell qPCR raw data Each data indicates the relative genes expression of VDR^−∕−^ and negative control (wild type).

10.7717/peerj.7230/supp-5 Data S2 Expression of VDR qPCR raw data Each data indicates the expression of VDR in different tissues at different period.

## Additional Information and Declarations

### Data Availability

The following information was supplied regarding data availability: The raw data is available in the Supplemental Files.
