# Supplementary material for: CRISPR/Cas9-mediated VDR knockout plays an essential role in the growth of dermal papilla cells through enhanced relative genes
Source: PeerJ. 2019 Jul 3;7:e7230. doi: 10.7717/peerj.7230 (PMC6612256; doi:10.7717/peerj.7230)
Supplement: Supplemental Information 1 — Supplementary 1. Sequence of VDR CDS and the sgRNA position. Supplementary 2. Sequence of negative cell control (wild type). [file peerj-07-7230-s001.docx]

**Supplementary 1. Sequence of *VDR* CDS and the sgRNA position.** The sequence of the VDR CDS is shown. sgRNA targeting site are shown in red and the PAM are shown in blue. Exon are showen in yellow. Forward and reverse primers are shown in green.

agtaaacgcactgttcagatgaagacagtgggatgcagagaggcaggacccctcacccaaaggagcccagcccataggcaccagacacggcctctggttctccgcgctttgacgccaggccacgccatgagtatcgccacagcgccccctgcagacgtgctccagcctgtctggggagacccgcttctgcttgtggccggcagcctcaggagggtgagctggtggcacccggaaccacaaagcgagggcgccgaggatcccactgaggcctgggccctggggacagctcgactttgctgagctcccggcagaagggctgccagcggcctcatccccgcctgctgttcttgcagccatggaggcgactgcggccagcacttccttacctgaccccggcgactttgaccggaacgtgccccggatctgcggggtgtgcggggaccgagccaccggcttccatttcaacgctatgacctgcgaaggctgcaaaggcttcttcaggtgagtcccctgcaagagcgaggagtagcgggtgaggaacagagtttcccgcaaagagaccctgcgtgttctgtgtctccttcctcaccaggcccagaaacactcccgttctcacagccacccgggccaacagggtctgtttcttcctcctctgccctcagctccagcttgtcatctgacccaactgcttcctgggtggaagcagttcatgtgaacccccatccgtccatcaccccttgacatacagccccctgcacccacccatgtccttccagagtgaacctcctaccattgatgatcagagcttcctagtccaggagactggcatggcctccaggaagcccaagagcttggcctcagagcacgcacccccaccctctcccctcctgccgccatcca

**Supplementary 2. Sequence of negative cell control (wild type).** sgRNA targeting site are shown in red and the PAM are shown in green. Base deletion are shown in blue dotted line. Forward and reverse primers are shown in green.

tggcacccggaaccacaaagcgagggcgccgaggatcccactgaggcctgggccctggggacagctcgactttgctgagctcccggcagaagggctgccagcggcctcatccccgcctgctgttcttgcagccatggaggcgactgcggccagcacttccttacctgaccccggcgactttgaccggaacgtgccccggatctgcggggtgtgcggggaccgagccaccggcttccatttcaacgctatgacctgcgaaggctgcaaaggcttcttcaggtgagtcccctgcaagagcgaggagtagcgggtgaggaa--gagtttcccgcaaagagaccctgcgtgttctgtgtctccttcctcaccaggcccagaaacact

cccgttctcacagccacccgggccaacagggtctgtttcttcctcctctg
