# Supplementary figures and images for: CRISPR/Cas9-mediated VDR knockout plays an essential role in the growth of dermal papilla cells through enhanced relative genes
Source: PeerJ. 2019 Jul 3;7:e7230. doi: 10.7717/peerj.7230 (PMC6612256; doi:10.7717/peerj.7230)

A

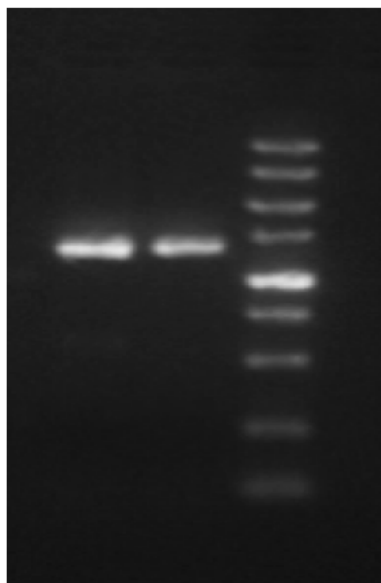

B

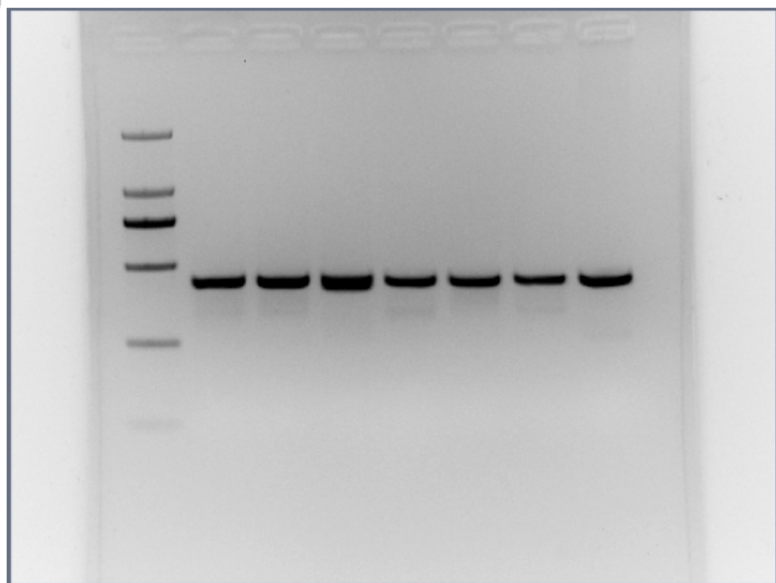

C

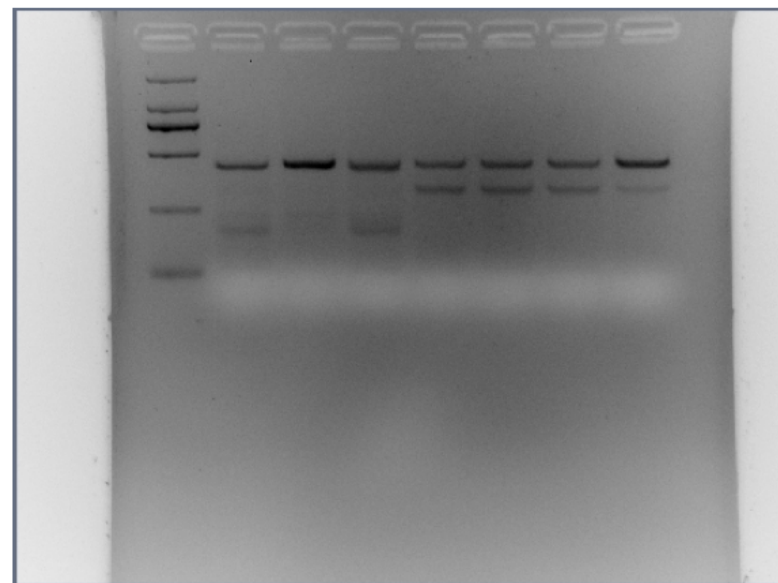

D

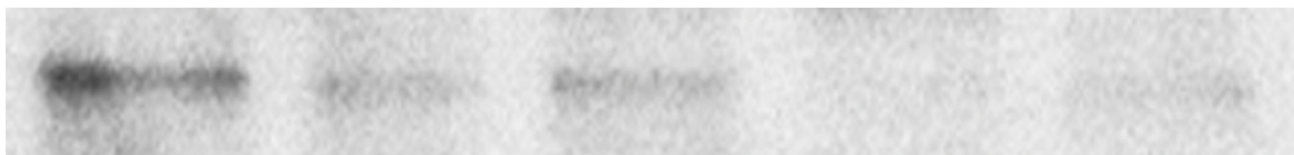

E

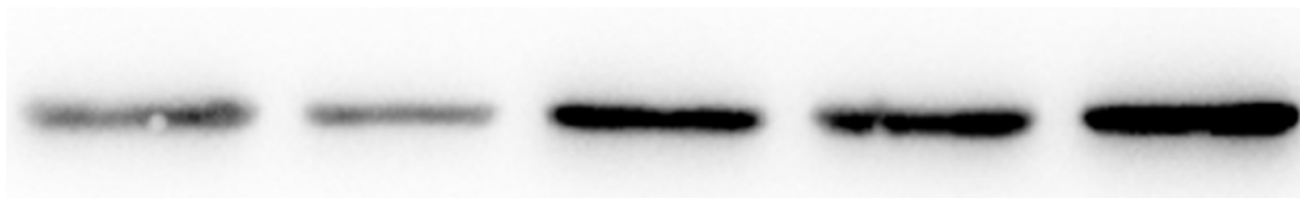

Supplement: Supplemental Information 3 — (A) Raw figure of VDR PCR product (5,000-bp DNA marker). (B) Raw figure of detection of sgRNA: Cas9-mediated cell DNA PCR products (2,000-bp DNA marker). (C) Raw figure of detection of sgRNA: Cas9-mediated cleavage by the T7E1 cleavage assay (2,000-bp DNA marker). (D) VDR western blot raw figure. (E) β-actin western blot raw figure. [file peerj-07-7230-s003.pdf]
